# 支气管肺泡灌洗液中免疫细胞亚群与晚期非小细胞肺癌一线免疫治疗疗效相关性的分析：一项病例对照研究

**DOI:** 10.3779/j.issn.1009-3419.2024.101.32

**Published:** 2024-12-20

**Authors:** Kai ZHANG, Liang SHI, Tongmei ZHANG, Li TONG, Song WEI, Hongxia LI

**Affiliations:** ^1^101149 北京，北京市结核病胸部肿瘤研究所/首都医科大学附属北京胸科医院肿瘤内科; ^1^Department of Medical Oncology, Beijing Tuberculosis & Thoracic Tumor Research Institute/Beijing Chest Hospital, Capital Medical University; ^2^首都医科大学基础临床联合实验室; ^2^Laboratory for Clinical Medicine, Capital Medical University, Beijing 101149, China

**Keywords:** 肺肿瘤, 肺泡灌洗液, 免疫治疗, 疗效预测, Lung neoplasms, Bronchoalveolar lavage fluid, Immunotherapy, Efficacy prediction

## Abstract

**背景与目的:**

免疫治疗已成为晚期非小细胞肺癌（non-small cell lung cancer, NSCLC）患者的标准治疗，但对于接受免疫治疗的晚期NSCLC患者仍缺乏特异性的疗效预测生物标志物，本研究通过探索支气管肺泡灌洗液（bronchoalveolar lavage fluid, BALF）中淋巴细胞亚群情况，并结合NSCLC患者的临床和实验室检查指标，探索潜在的免疫治疗相关性生物标志物。

**方法:**

回顾性分析2020年3月至2022年11月在首都医科大学附属北京胸科医院进行电子支气管镜检查并接受一线免疫治疗的82例局部晚期或晚期NSCLC患者的资料，通过Logistic回归和随机森林模型评估BALF中免疫细胞亚群与疗效的关联，并验证预测模型的敏感性。

**结果:**

入组患者均接受一线免疫治疗，按照临床诊疗规范进行疗效评价：纳入的82例患者中，48例达到客观缓解，34例未达到。对采集的相关指标与临床治疗最佳疗效之间的关系进行分析，结果显示BALF中的总淋巴细胞百分比升高与较好的免疫治疗疗效有关（*P*<0.05），BALF中的辅助性T细胞百分比的升高与免疫治疗的疗效不佳有关（*P*<0.05）。

**结论:**

BALF中总淋巴细胞和辅助性T细胞比例可作为晚期NSCLC免疫治疗疗效的重要预测指标，联合其他指标构建的多变量模型可以进一步提高预测准确性。

## Abstract (en)

**Background and objective:**

Immunotherapy has been a standard treatment for the patients with advanced non-small cell lung cancer (NSCLC), however, reliable biomarkers for predicting the response remain unclear. This study explores the subpopulations of lymphocytes in bronchoalveolar lavage fluid (BALF) and combines clinical and laboratory examination indicators of NSCLC patients to identify potential biomarkers related to immunotherapy.

**Methods:**

A retrospective analysis was conducted on 82 patients with locally advanced or metastatic NSCLC who underwent electronic bronchoscopy and received first-line immunotherapy at Beijing Chest Hospital, Capital Medical University between March 2020 and November 2022. Logistic regression and random forest models were employed to determine the correlation between immune cell subsets in BALF and response of immunotherapy. The predictive value was validated by the model.

**Results:**

All patients enrolled received first-line immunotherapy, and the efficacy was evaluated according to clinical guidelines: among the 82 patients included, 48 patients got objective response and the other 34 did not achieve. The relationship between collected indicators and the best clinical treatment response was analyzed. The result shows that a higher percentage of total lymphocytes in BALF was associated with good response of first-line immunotherapy (*P*<0.05), while a higher percentage of T helper cells in BALF was associated with poor prognosis (*P*<0.05).

**Conclusion:**

The proportions of total lymphocytes and T helper cells in BALF could be used as predictive biomarkers for first-line immunotherapy in late stage NSCLC. A multivariable model improves predictive accuracy.

肺癌是我国恶性肿瘤相关死亡的首要原因^[1]^。根据病理类型，肺癌主要分为两类：非小细胞肺癌（non-small cell lung cancer, NSCLC）和小细胞肺癌（small cell lung cancer, SCLC），其中NSCLC约占确诊肺癌的85%^[2]^。相当一部分NSCLC患者就诊时已处于晚期，预后较差，免疫治疗对于驱动基因阴性晚期NSCLC的治疗具有重要意义。但是目前免疫治疗在NSCLC中仅能使部分患者获得生存受益^[3]^。对NSCLC晚期患者免疫治疗效果的预测，目前还是一个临床难题。

对生物标志物分析仍然是预测NSCLC疗效的研究热点^[4,5]^。目前用于预测免疫治疗疗效的生物标志物如肿瘤突变负荷（tumor mutational burden, TMB）、组织基因表达谱等指标都需要获取肿瘤组织以进行进一步操作，获取肿瘤组织存在一定困难、成本较高且很难动态监测。相比之下，电子支气管镜检查是确诊肺癌的重要临床手段，支气管肺泡灌洗是支气管镜检查中的常规操作，由于其获得的支气管肺泡灌洗液（bronchoalveolar lavage fluid, BALF）中包含肿瘤局部的免疫细胞、肿瘤细胞及相关DNA、RNA，因此可为肺癌诊断和鉴别诊断提供重要依据^[6]^。但是，当前缺乏利用BALF相关指标用于晚期NSCLC免疫治疗疗效的相关性研究报道。本研究对局部晚期或晚期NSCLC接受免疫治疗患者的BALF的相关结果进行回顾性分析，结合其他临床特点及实验室相关化验结果，探索预测免疫治疗疗效组合，以期为局部晚期或晚期NSCLC免疫治疗疗效预测和预后评估提供新的方法和手段。

## 1 资料与方法

### 1.1 一般资料

回顾性纳入2020年3月至2022年11月在首都医科大学附属北京胸科医院接受电子支气管镜检查，并接受一线免疫治疗的不可手术及根治性放疗的局部晚期或晚期NSCLC患者82例（图1），所有患者均接受一线化疗联合免疫检查点抑制剂治疗。所有资料均来源于首都医科大学附属北京胸科医院数据库。纳入标准：（1）经病理学诊断证实，采用国际肺癌研究会（International Association for the Study of Lung Cancer, IASLC）NSCLC第8版分期诊断为不可手术切除及根治性放疗的局部晚期（IIIB-IIIC期）或晚期（IV期）患者；（2）能够接受气管镜诊断检查；（3）组织学检测表皮生长因子受体（epidermal growth factor receptor, EGFR）/间变性淋巴瘤激酶（anaplastic lymphoma kinase, ALK）/c-ros肉瘤致癌因子-受体酪氨酸激酶（ROS proto-oncogene 1, receptor tyrosine kinase, ROS1）基因改变阴性；（4）患者接受一线化疗联合免疫治疗；（5）年龄≥18岁；（6）能够依从治疗方案；（7）东部肿瘤协作组体能状态（Eastern Cooperative Oncology Group performance status, ECOG PS）评分0-2分；（8）预期生存期≥12周；（9）足够的血液学功能（包括血常规、肝功能、肾功能及电解质）；（10）理解并自愿签署书面知情同意书。此外，符合以下任何条件的患者均被排除：（1）既往存在合并的自身免疫性疾病史：包括并不限于系统性红斑狼疮、自身免疫性血管炎、自身免疫性肾炎等；（2）既往患有其他恶性肿瘤病史；（3）既往患有需要激素治疗的放射性肺炎、间质性肺疾病、药物诱导的间质性肺疾病或任何具临床证据的活动性间质性肺疾病；（4）既往或现在患有严重的或不能控制的全身性疾病（包括不稳定或不能代偿的心脏、呼吸、肝或肾脏疾病等）的证据；（5）患有不稳定的系统性疾病（如四级高血压、不稳定心绞痛、充血性心力衰竭、肝肾疾病、活动性感染：肺炎/结核病/病毒性肝炎/梅毒/艾滋病等）；（6）明确的精神或神经障碍史，比如癫痫或痴呆等；（7）孕妇和哺乳期女性；（8）研究者认为不宜参加本课题者。本研究方案经首都医科大学附属北京胸科医院伦理委员会审批（编号：LW-2024-007）。

**图 1:**
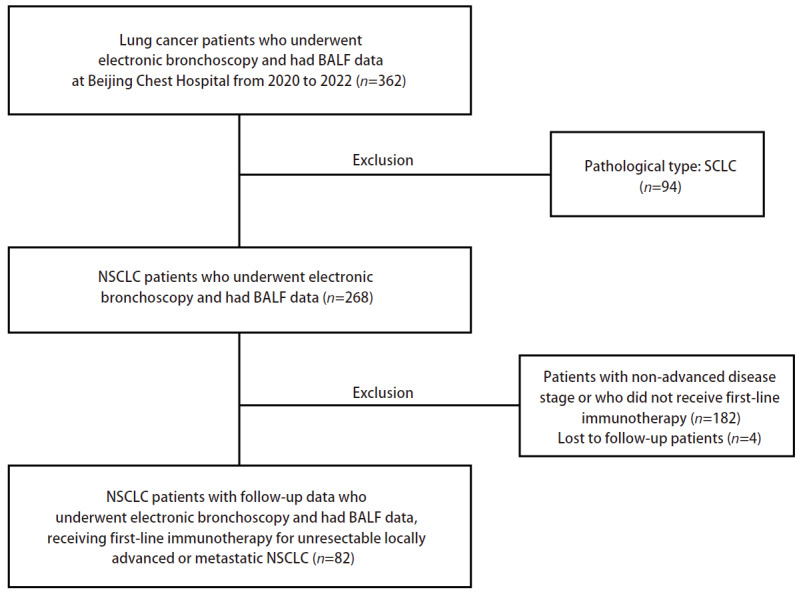
研究队列纳入流程图

### 1.2 资料收集

对于所有符合纳入标准的患者，收集患者基本疾病信息、临床病理学资料及相关化验指标，包括：性别、吸烟史、肿瘤组织类型、细胞程序性死亡配体1（programmed cell death ligand 1, PD-L1）肿瘤细胞阳性比例评分（tumor proportion score, TPS）、外周血白介素-6（interleukin-6, IL-6）、IL-8水平、外周血免疫细胞亚群和BALF免疫细胞亚群、治疗前白细胞总数、中性粒细胞、血小板计数、乳酸脱氢酶等。治疗反应根据实体瘤疗效评价标准（Response Evaluation Criteria in Solid Tumors, RECIST）1.1版进行疗效评价，在治疗后按照临床实践标准进行随访以评估抗肿瘤治疗疗效，末次随访时间为2023年6月。具体标准为：（1）完全缓解（complete response, CR）：靶病灶完全消退；（2）部分缓解（partial response, PR）：总靶病灶减少30%以上；（3）疾病稳定（stable disease, SD）：总靶病灶减少少于30%或增加少于20%；（4）疾病进展（progressive disease, PD）：总靶病灶增加20%以上。以一线治疗的最佳疗效作为疗效记录进行分析评估。

### 1.3 统计学方法

为比较不同疗效组别间指标的差异，分类计数资料通过例数（%）表示并采用卡方检验；定量数据通过均数±标准差（Mean±SD）或中位数（Q1, Q3）表示，其中正态分布数据采用t检验、非正态分布数据采用Wilcoxon秩和检验分析组间差异。根据KEYNOTE-001和KEYNOTE-042临床试验确定PD-L1 TPS=50%为最佳截断值^[7⇓-9]^。通过Logistic回归分析，将单因素分析中与患者的治疗效果有关联的因素（*P*<0.1）纳入多因素Logistic回归分析并确定患者BALF生物标志物中对免疫治疗疗效有影响的因素，同时通过比较受试者工作特征（receiver operating characteristic, ROC）的曲线下面积（area under the curve, AUC）和随机森林模型来验证有关联的因素的预测敏感性。本研究所有的统计学分析及绘图采用R 4.3.1版软件与GraphPad Prism 9软件进行。涉及的R语言包包括：“moonBook”“rms”“forestmodel”“pROC”“randomForest”“dplyr”“ggpubr”。*P*<0.05为差异具有统计学意义。

## 2 结果

### 2.1 患者临床、病理特征及疗效评价分组

本研究纳入82例接受一线免疫治疗的局部晚期或晚期NSCLC患者。患者基线临床特征详见表1。其中性别以男性为主（96.34%）。74例（90.24%）吸烟，8例（9.76%）不吸烟。PD-L1阳性表达低水平组58例（70.73%）、表达高水平组24例（29.27%）。82例NSCLC患者中，最常见的病理类型为鳞状细胞癌（73.17%），非鳞状细胞癌22例（26.83%）。两组组间比较BALF中辅助性T细胞百分比的差异有统计学意义（*P*=0.044），BALF中其他免疫细胞亚群的两组间差异无统计学意义（*P*>0.05）（图2）。本研究对所纳入的NSCLC患者在接受一线免疫治疗后规律进行影像学评价，纳入患者的中位随访治疗周期数为6个周期。根据客观缓解率（objective response rate, ORR）中临床疗效的定义，PR+CR（*n*=48）定义为客观缓解，PD+SD（*n*=34）定义为未达客观缓解。其中，有2例患者达到CR，46例患者达PR。有7例患者评价为PD，有27例患者评价为SD。

**表 1:** 患者基线特征

Variables	Total (*n*=82)	PR+CR cohort (*n*=48)	PD+SD cohort (*n*=34)	P
Basic information				
Gender				0.567
Male	79 (96.34%)	47 (97.92%)	32 (94.12%)	
Female	3 (3.66%)	1 (2.08%)	2 (5.88%)	
Age (yr)	63.52±8.38	65.23±8.38	61.12±7.87	0.028
Smoking history				0.713
Never	8 (9.76%)	4 (8.33%)	4 (11.76%)	
Current and former	74 (90.24%)	44 (91.67%)	30 (88.24%)	
Histology				0.848
LUSC	60 (73.17%)	36 (75.00%)	24 (70.59%)	
Non-LUSC	22 (26.83%)	12 (25.00%)	10 (29.41%)	
Location				0.230
Central type	75 (91.46%)	42 (87.50%)	33 (97.06%)	
Peripheral type	7 (8.54%)	6 (12.50%)	1 (2.94%)	
PD-L1				0.028
TPS<50%	58 (70.73%)	29 (60.42%)	29 (85.29%)	
TPS≥50%	24 (29.27%)	19 (39.58%)	5 (14.71%)	
Peripheral blood test indicators				
Lymphocyte (%)	19.08±7.58	19.69±7.57	18.23±7.64	0.393
T cell (%)	68 (61, 79)	72 (62, 80)	67 (60, 75)	0.189
Th cell (%)	42.29±10.79	43.67±10.17	40.34±11.48	0.170
Treg (%)	8 (6, 11)	8 (6, 11)	7 (5, 10)	0.057
NK cell (%)	18 (11, 27)	17 (11, 26)	19 (13, 27)	0.621
NK-T cell (%)	6 (3, 30)	6 (3, 38)	6 (3, 9)	0.405
Th cell/CD8 T cell	2 (1, 2)	2 (1, 2)	2 (1, 2)	0.672
Monocyte (%)	7 (6, 10)	8 (6, 10)	7 (5, 10)	0.489
Activated T cell (%)	15 (10, 26)	13 (11, 23)	18 (9, 28)	0.672
IL-6 (pg/mL)	7 (3, 14)	7 (3, 14)	7 (3, 17)	0.742
IL-8 (pg/mL)	9 (4, 18)	8 (4, 17)	13 (5, 36)	0.139
IFN-λ (pg/mL)	2 (1, 4)	2 (1, 4)	3 (1, 4)	0.292
White blood cell (×10^9^/L)	8 (6, 9)	7 (6, 9)	8 (6, 10)	0.228
Neutrophil (×10^9^/L)	5 (4, 6)	5 (4, 6)	5 (4, 7)	0.131
PLT (×10^9^/L)	259 (217, 339)	250 (211, 279)	295 (237, 379)	0.028
LDH (U/L)	162 (145, 207)	162 (147, 203)	162 (145, 224)	0.770
Subsets of BALF immune cells				
Lymphocytes (%)	5 (3, 8)	6 (3, 10)	4 (2, 7)	0.075
T cell (%)	62 (57, 77)	62 (57, 77)	65 (55, 80)	0.585
Th cell (%)	22 (15, 31)	19 (13, 27)	26 (17, 35)	0.044
Treg (%)	11 (7, 18)	10 (7, 19)	12 (6, 18)	0.929
NK cell (%)	9 (5, 18)	9 (5, 18)	11 (5, 20)	0.522
NK-T cell (%)	12 (6, 24)	14 (6, 28)	10 (6, 17)	0.243
Th cell/CD8 T cell	1 (0, 2)	1 (0, 2)	1 (0, 2)	0.679
Monocyte (%)	7 (3, 15)	8 (4, 13)	6 (3, 16)	0.877
Activated T cell (%)	22 (15, 35)	24 (15, 39)	19 (13, 31)	0.347

Categorical count data were expressed as the number of cases (%) and quantitative data were expressed as Mean±standard deviation (Mean±SD) or median (Q1, Q3). LUSC: lung squamous cell carcinoma; PD-L1: programmed cell death ligand 1; TPS: tumor proportion score; Th cell: T helper cell; Treg: regulatory T cell; NK cell: natural killer cell; IL-6: interleukin-6; IL-8: interleukin-8; IFN-λ: interferon-λ; PLT: platelet; LDH: lactate dehydrogenase; BALF: bronchoalveolar lavage fluid; PR: partial response; CR: complete response; PD: progressive disease; SD: stable disease.

**图 2:**
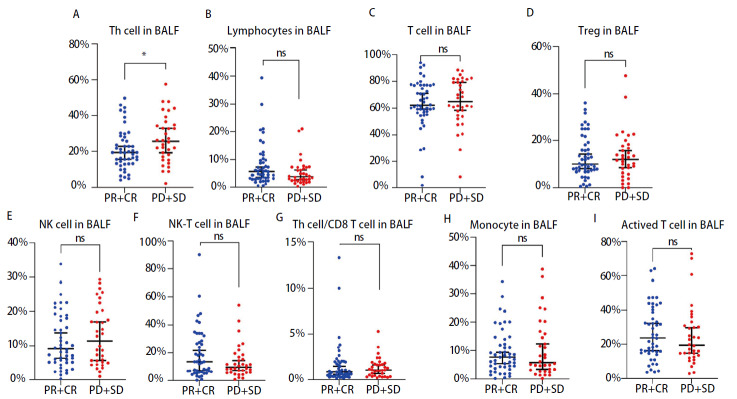
BALF中免疫细胞亚群在两个不同疗效组间示意图。A：BALF中辅助性T细胞百分比在两个不同疗效组间的差异有统计学意义；B-I：在BALF中，总淋巴细胞（B）、T细胞（C）、调节性T细胞（D）、自然杀伤细胞（E）、自然杀伤T细胞（F）、辅助性T细胞/CD8 T细胞比值（G）、单核细胞（H）及活化T细胞（I）的百分比在两个不同疗效组间的差异均无统计学意义。*: *P*<0.05.

### 2.2 晚期NSCLC一线免疫治疗疗效相关影响因素的探索

采用Logistic回归分析对31项临床及病理学资料进行筛选。通过单因素Logistic回归分析（表2）发现，年龄、肿瘤组织中PD-L1、外周血中调节性T细胞百分比、外周血中IL-8水平、治疗前外周血中性粒细胞总数、外周血中血小板计数、BALF中总淋巴细胞百分比以及BALF中辅助性T细胞百分比，以上8个指标可能与患者免疫治疗疗效有关联（*P*<0.1）。将以上指标纳入多因素Logistic回归分析拟合模型，确定了4个对晚期NSCLC免疫治疗疗效有影响的独立预测因子：年龄（*P*=0.021）、肿瘤组织中PD-L1表达水平（*P*=0.008）、BALF中总淋巴细胞百分比（*P*=0.042）以及BALF中辅助性T细胞百分比（*P*=0.048）。结果表明，对于局部晚期及晚期NSCLC的一线免疫治疗，BALF中辅助性T细胞百分比的升高与免疫治疗疗效不佳有关联；年龄大、肿瘤组织中PD-L1水平高以及BALF中总淋巴细胞百分比高对免疫治疗的有效性有影响（图3）。

**表 2:** 单因素Logistic回归分析

Variables	OR	95%CI	P
Basic information			
Gender	2.94	0.27-64.78	0.387
Age	0.94	0.88-0.99	0.032
Smoking history	0.68	0.15-3.09	0.608
Histology	1.25	0.46-3.36	0.657
Location	4.71	0.75-91.36	0.160
PD-L1	0.26	0.08-0.75	0.019
Peripheral blood test indicators			
Lymphocyte	0.97	0.92-1.03	0.389
T cell	0.98	0.94-1.01	0.196
Th cell	0.97	0.93-1.01	0.171
Treg	0.87	0.74-1.01	0.073
NK cell	1.00	0.96-1.05	0.888
NK-T cell	0.98	0.95-1.00	0.111
Th cell/CD8 T cell	1.01	0.64-1.58	0.952
Monocyte	0.99	0.88-1.10	0.836
Activated T cell	1.02	0.99-1.05	0.227
IL-6	1.00	0.99-1.00	0.658
IL-8	1.02	1.00-1.04	0.090
IFN-λ	0.98	0.84-1.12	0.794
White blood cell	1.18	0.97-1.47	0.114
Neutrophil	1.24	0.99-1.61	0.084
PLT	1.00	1.00-1.01	0.055
LDH	1.00	1.00-1.01	0.253
Subsets of BALF immune cells			
Lymphocytes	0.93	0.84-1.00	0.093
T cell	1.00	0.98-1.03	0.710
Th cell	1.04	1.02-1.08	0.045
Treg	1.00	0.95-1.05	0.995
NK cell	1.02	0.97-1.08	0.388
NK-T cell	0.97	0.94-1.00	0.112
Th cell/CD8 T cell	0.91	0.66-1.16	0.488
Monocyte	1.01	0.96-1.06	0.623
Activated T cell	0.99	0.96-1.02	0.588
OR: odds ratio.			

**图 3:**
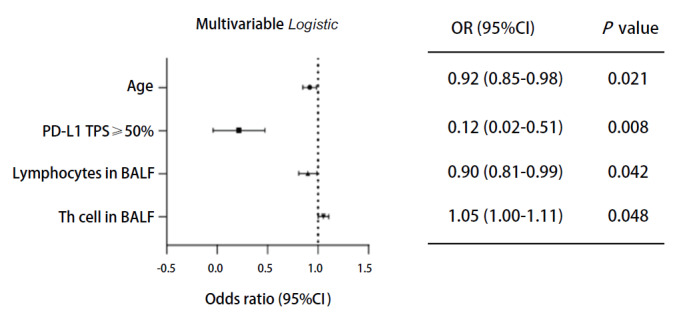
多因素Logistic回归分析结果的森林图

### 2.3 不同指标的预测价值评价

NSCLC的免疫治疗疗效与患者的免疫状态及微环境关系密切，年龄可能并不能直接反映这些关键的生物学机制，因此在利用不同指标评价其对于免疫治疗的预测价值时未纳入年龄这一人口学变量。如图4所示，使用AUC来评估不同变量在预测一线免疫治疗疗效时的能力，当联合使用BALF中总淋巴细胞百分比、BALF中辅助性T细胞百分比和PD-L1表达水平构建多变量模型时，ROC曲线的AUC为0.752。相比之下，单独使用肿瘤组织PD-L1表达水平、BALF中辅助性T细胞百分比和BALF中总淋巴细胞百分比进行预测时，AUC分别为0.624、0.631和0.616。通过比较这些ROC曲线的AUC发现，多变量模型的AUC与单独使用BALF中辅助性T细胞百分比（*P*=0.045）和BALF中总淋巴细胞百分比（*P*=0.028）的模型相比差异具有统计学意义，与单独使用PD-L1表达水平的模型（*P*=0.084）相比具有边际显著性。BALF中的总淋巴细胞百分比和辅助性T细胞百分比是与NSCLC免疫治疗疗效具有关联的预测指标，且综合使用BALF中总淋巴细胞百分比、BALF中辅助性T细胞百分比和PD-L1表达水平3个指标预测NSCLC免疫治疗疗效的效能高于仅单独使用其中任何一个单一指标进行预测。

**图 4:**
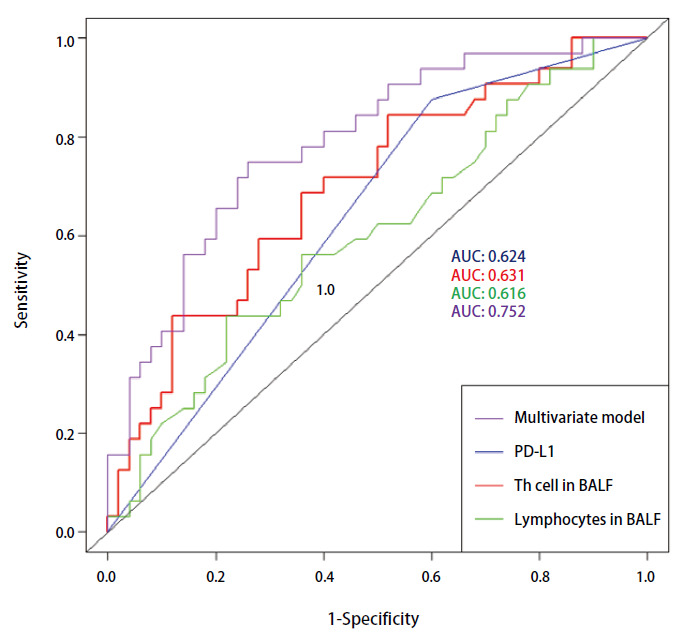
不同指标用于预测晚期NSCLC一线免疫治疗有效性的ROC曲线

### 2.4 预测指标的敏感性验证

对于上述指标的预测价值评价，还采用随机森林模型来对上述筛选预测指标进行验证。纳入包括外周血免疫细胞亚群和BALF免疫细胞亚群在内的31项临床病理学资料，评价其与晚期NSCLC一线免疫治疗疗效的相关性。在随机森林模型中，使用了1000棵决策树来预测目标变量。通过设置随机种子确保结果的可重复性，并在模型训练过程中计算了每个变量在节点分裂过程中的节点增量纯度值，以评估各变量对模型预测性能的贡献。节点增量纯度值表示在使用特定变量进行节点分裂时模型纯度提升的程度，这一指标越高，说明该变量在决策过程中越为重要。

采用排列特征重要性法对影响治疗疗效的变量进行排序，节点增量纯度值排在前5位的变量分别为：血小板、BALF中辅助性T细胞百分比、乳酸脱氢酶、外周血中调节性T细胞百分比、BALF中总淋巴细胞百分比。这一结果也验证了在BALF免疫细胞亚群中，总淋巴细胞百分比和辅助性T细胞百分比这两个变量在决策树的节点分裂中起着关键作用，显著提升了模型的预测性能，表明了它们在预测一线免疫治疗疗效中的重要影响（图5）。而此结果也与多因素Logistic回归的结果高度一致，进一步证实了BALF中局部免疫状态在预测NSCLC免疫治疗疗效中的重要性。

**图 5:**
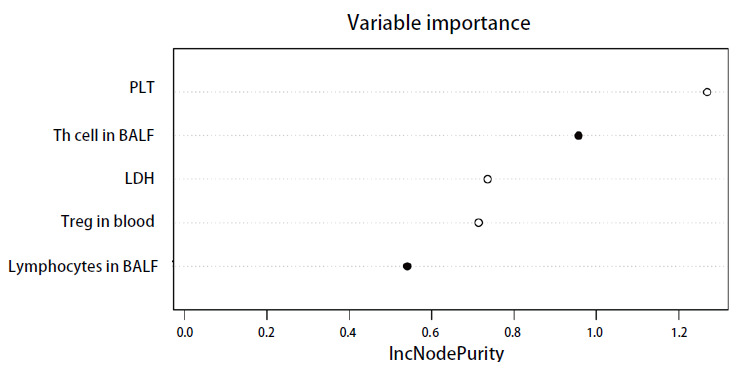
随机森林模型图。该图表展示了在随机森林模型中主要变量的节点增量纯度值，表明了它们对模型预测性能的贡献。高纯度值意味着该变量在提高模型的分类准确性方面起到了关键作用。

## 3 讨论

对于不可手术及根治性放疗的局部晚期及晚期NSCLC主要依赖于全身治疗，免疫联合化疗或免疫单药治疗已经是当前的标准一线治疗^[7,10⇓-12]^。以PD-1/PD-L1单抗为代表的免疫检查点抑制剂已被证实可以改善晚期NSCLC患者的生存，国内已批准多个PD-1/PD-L1抑制剂用于晚期NSCLC的治疗^[13]^。既往研究^[10,14]^显示，肿瘤组织中PD-L1表达水平可作为疗效预测的重要指标，高PD-L1表达（≥50%）的晚期NSCLC患者可能更有望从PD-1/PD-L1抑制剂治疗中获益，KEYNOTE-001研究^[9]^的数据表明，对于PD-L1 TPS≥50%的晚期NSCLC患者，无论是先前接受治疗（*P*<0.001）还是先前未接受过治疗（*P*=0.01），其对帕博利珠单抗产生的应答率均超过了PD-L1 TPS为1%-49%和<1%的患者。KEYNOTE-010研究^[15]^的数据表明，对于PD-L1 TPS≥50%的晚期NSCLC患者，使用免疫治疗与单纯化疗相比，可以明显延长患者无进展生存期（progression-free survival, PFS）（*P*<0.001）。本研究分析结果也显示PD-L1 TPS≥50%的晚期NSCLC患者能够收获更好的免疫治疗疗效（*P*=0.006）。但是仍然缺乏更为有效的预测疗效指标，从而更加准确地识别可能治疗失败的人群，加强治疗手段，提高治疗效果。

既往研究^[16,17]^大多关注于外周血液中生物标志物与免疫治疗效果的相关性，常规的外周血检查一般仅能评估全身整体的免疫状态，但不能反映局部的免疫状态，BALF可以作为大量局部免疫细胞和介质的来源用以分析局部免疫状态。由于流式细胞术等精确的鉴定方法，使得这些细胞进行深度的表型分析成为可能^[18,19]^。目前已有研究^[20,21]^显示肺癌患者和健康志愿者的BALF在免疫细胞库方面存在差异。近期也有研究^[22]^关注到免疫治疗过程中BALF指标的动态变化，提示BALF中的免疫细胞亚群与外周血免疫细胞亚群相比，CD4^+^/CD8^+^ T细胞比例明显降低，BALF和外周血细胞谱之间存在显著差异。同样，在本研究的队列中，BALF中总淋巴细胞比例与外周血总淋巴细胞比例之间相关性较低，进一步提示外周血不能完全代表局部免疫状态，BALF的免疫微环境可能具有独特特征。BALF的灌洗、回收过程以及本研究中对于肿瘤组织在解剖学中的位置的分析，提示BALF对于肺癌的局部微环境反应具有很好的特异性。本研究收集了NSCLC患者的临床及病理学资料，结合临床常规的BALF免疫细胞亚群检测结果，探索与接受一线免疫治疗疗效相关的因素，预测一线免疫治疗的疗效，为临床治疗决策提供帮助。

当然本次研究仍然存在一定的局限性。首先，研究采用的是回顾性研究且使用的是单中心的数据集，入组的患者数量相对较少，这可能会限制我们的研究结果的普遍性。在未来的研究中，课题组将进一步收集多中心数据，继续扩大样本量，实现预测模型建立及训练集和验证集的拆分，使本次分析结果比目前的研究更可靠且更具临床应用性。其次，研究对于治疗效果为SD和PD患者合并分析的分组策略，主要基于ORR这一二分类指标的设计，旨在简化对治疗有反应和无反应患者的分类。对于CR和PR的患者采取标准治疗，而对于预期疗效无反应（SD+PD）的患者可能考虑采取更强的联合治疗模式，从而获得更好的治疗反应率。当然尽管ORR反映了抗肿瘤治疗的短期治疗有效率，但在长期生存预后方面存在局限性。鉴于此，课题组计划在未来开展前瞻性的队列研究，并纳入PFS和总生存期（overall survival, OS）等生存时间指标，以提供更全面的疗效评价。这不仅有助于更好地理解免疫治疗的长期效果，还能为临床实践提供更有价值的指导。

综上所述，本研究初步确定了在BALF免疫细胞亚群中，总淋巴细胞百分比升高预示着NSCLC免疫治疗更好的疗效，辅助性T细胞百分比的升高则预示着NSCLC免疫治疗有效的可能降低。结合患者肿瘤组织PD-L1表达水平与BALF中的T细胞亚群指标构建的多因素预测模型可以更加有效地预测晚期NSCLC患者一线免疫治疗的有效性。当然，未来需要进一步前瞻性研究阐明这些生物标志物的价值。期待这些BALF免疫细胞亚群数据可以帮助临床医生更精准地预测一线免疫治疗晚期NSCLC的疗效，从而为患者制定更加精准的治疗策略。
